# Spatial Multi-Omics Analysis of the Qianqiu Goat Gut Microbiome and Metabolome

**DOI:** 10.3390/ijms262411815

**Published:** 2025-12-07

**Authors:** Panpan Guo, Wenjuan Qin, Wencheng Song, Hongquan Chen

**Affiliations:** 1 School of Animal Science and Technology, Anhui Agricultural University, Hefei 230036, China; guopp@iim.ac.cn (P.G.); qinwenjuan0611@gmail.com (W.Q.); 2 Hefei Institute of Physical Science, Chinese Academy of Sciences, Hefei 230031, China; wencsong@cmpt.ac.cn; 3 Key Laboratory of Anhui Local Livestock and Poultry Genetic Resources Conservation and Biobreeding of Anhui Province, Hefei 230036, China

**Keywords:** integrated analysis, metabolomics, microbiome, Qianqiu goat, spatial multi-omics

## Abstract

This study profiled the rumen (RM), small intestine (SI), and large intestine (LI) of 24 samples collected from eight 6-month-old Qianqiu goats (body weight 28.40 ± 1.80 kg), with the samples equally divided into three groups. A combination of methods was used, including 16S rRNA sequencing, untargeted liquid chromatography–mass spectrometry (LC-MS) metabolomics, Orthogonal Partial Least Squares Discriminant Analysis (OPLS-DA), Kyoto Encyclopedia of Genes and Genomes (KEGG) enrichment, and weighted gene co-expression network analysis-based module detection (WGCNA) with network integration. An uncommon composition of organisms dominated the SI: the hydrogenotrophic methanogens Methanobrevibacter (SI 24.51%; RM 1.92%; LI 2.19%) and Methanosphaera (SI 0.43%; RM 0.02%; LI 0.02%), together with the acetogen Acetitomaculum (SI 1.58%; RM 0.34%; LI 0.11%), were markedly more abundant compared to the RM or LI. Correlation and pathway analyses indicated that Methanobrevibacter was positively correlated with a steroid-type lipid metabolite (*r* = 0.52, *p* < 0.05) and with bile-acid-related metabolites. Acetitomaculum was positively correlated with several metabolites: 4-Hydroxyphenyl 4-hydroxybenzoate (r = 0.79, *p* < 0.05), 2-Aminoethyl dihydrogen phosphate (*r* = 0.76, *p* < 0.05), 1-Myristoyl-2-stearoyl-sn-glycero-3-phosphocholine (*r* = 0.76, *p* < 0.05), and 1,2-Dioleoyl-sn-Glycero-3-Phosphocholine (*r* = 0.74, *p* < 0.05). Together, these data define a small-intestinal microbial–metabolite module in Qianqiu goats characterized by elevated abundances of specific methanogens and acetogens in the SI. Specific positive correlations were identified between these taxa and metabolites associated with lipids and bile acids.

## 1. Introduction

The four-chambered digestive system of ruminants has long been a subject of interest for researchers. This system efficiently converts low-nutrient plant material into energy and proteins, adapting surrounding life forms to the ruminant’s environment [1]. It offers an insight into the study of symbiotic relationships, biological conversion efficiency, and evolutionary intelligence [2]. From the anatomical perspective, the rumen, a unique fermentative organ in ruminants, converts fiber-rich substances into volatile fatty acids through microbial action [3]. The small intestine primarily relies on enzymatic digestion and active absorption, while the large intestine plays a crucial role in maintaining electrolyte balance [4,5]. At the microbial level, differences in pH, oxygen concentration, and nutrient substrates across various gastrointestinal (GI) segments lead to significant diversification in microbial communities and metabolic pathways. Metabolites often serve as a “chemical language” between the host and microbe, regulated by the host’s genetic makeup. This, in turn, influences microbial community dynamics [6,7]. For instance, the accumulation of short-chain fatty acids (SCFAs) in the rumen can modulate host energy metabolism. In the intestine, bile acid metabolism influences immune homeostasis via the Farnesoid X receptor (FXR) signaling pathway [8,9].

The Qianqiu goat is a newly recognized, Anhui-endemic local germplasm (China), distributed mainly in Tianchang City and Laian County, Anhui Province. It is white-coated, and its horns are typically counter-spiral or arched (Supplementary Figure S1). In the measured cohort, adult males averaged at 60.3 kg and females averaged at 44.4 kg in live body weight; the mean dressing percentage was 53.71% and meat yield was 48.03%. Taken together, this breed’s narrow geographic range and distinctive phenotype provide a biological context for the putative metabolic adaptations observed. Data are taken from our measured cohort. To date, no public literature reports have been made for these values in Qianqiu goats.

Therefore, systematic studies on metabolic heterogeneity in the Qianqiu goat remain limited, particularly regarding the mechanisms that link multi-segment metabolic profiles to microbial functions. Current research on local goats predominantly focuses on genetic improvement and production performance, while systematic microbial analyses remain in their infancy [10,11]. Additionally, traditional culture-dependent methods are limited by the low cultivability of gut microbes (only approximately 20% can be cultured in vitro), leaving the functional potential of many uncultured microbes unexplored [12]. Because small input samples can yield high-resolution data via amplicon sequencing, this bottleneck can be overcome. However, applying this method to complex, multi-segment GI-tract analysis remains underdeveloped [13]. In addition, metabolomics has also been used to study microbial function and host genetic characteristics. Metabolomics, a branch of systems biology, uses high-throughput metabolite detection to provide a comprehensive understanding of metabolic network changes under physiological or pathological conditions [14].

There have been significant improvements in metabolite identification due to advances in technologies such as liquid chromatography–mass spectrometry (LC–MS) and nuclear magnetic resonance. This has enabled unbiased identification in complex biological samples, such as GI contents [15]. In research, metabolomics has been successfully applied to regulate rumen fermentation, mitigate methane emissions, and enhance nutrient utilization efficiency [16]. For example, studies have identified significant correlations between specific metabolic pathways and methane production in sheep under different feeding regimes [17]. Another study revealed a potential link between polyamine metabolites and intestinal barrier function in goats [18,19].

Despite some progress in the study of ruminant GI metabolism, research on the Qianqiu goat remains limited for the following reasons: (1) most current metabolomic data are based on Western commercial breeds, neglecting the unique metabolic adaptations of local breeds developed through long-term natural selection [20]; (2) many studies adopt a whole-metabolome approach, failing to analyze spatiotemporal differences in metabolic functions across different GI segments [21]; (3) the identification of differential metabolites often remains at a descriptive statistical level, lacking integrative analysis with microbiome data, which hinders in-depth exploration of the mechanisms driving metabolic heterogeneity [22].

Additionally, because the Qianqiu goat lives year-round in low-altitude, low-nutrient environments, its GI metabolic system may have evolved unique energy utilization strategies and stress-response pathways that have yet to be fully explored [23].

In this study, we combined amplicon sequencing with metabolomics to investigate microbiome composition and metabolic profiles across the rumen, small intestine, and colon of Qianqiu goats. Specifically, we aimed to address the following questions: (1) Are there statistically significant differences in the microbial community composition across different GI segments? (2) Does the microbial symbiotic network exhibit a modular structure, and are key keystone taxa associated with host metabolic phenotypes?

## 2. Results

### 2.1. Operational Taxonomic Unit (OTU) Clustering Analysis

**Figure 1:**
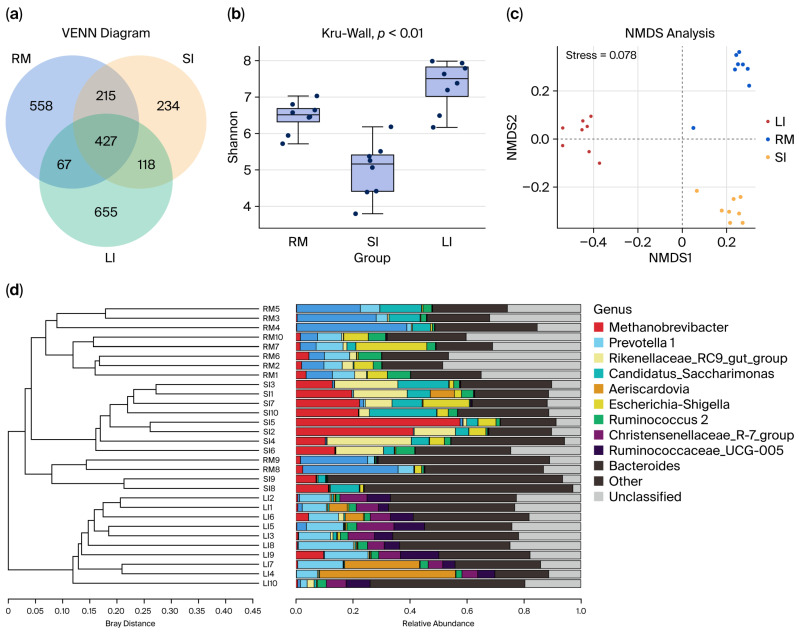
(**a**) Venn diagram and UpSet plot illustrating shared and unique bacterial operational taxonomic units (OTUs) among the rumen (RM), small intestine (SI), and large intestine (LI) chyme samples. (**b**) Shannon diversity index of bacterial communities in chyme samples from RM, SI, and LI. (**c**) Non-metric multidimensional scaling (NMDS) plot based on Bray–Curtis distance, showing the clustering patterns of microbial communities across the RM, SI, and LI. (**d**) Hierarchical tree illustrating the unweighted pair group method with arithmetic mean (UPGMA) clustering results based on microbial community composition in the three GI regions. In (**b**), dots indicate individual samples (Shannon diversity values) overlaid on the boxplots.

Clustering effective bacterial tag sequences at 97% sequence identity yielded 2284 OTUs. Venn analysis (Figure 1a) revealed that 427 OTUs (18.7% of the total) were shared among all three GI segments. Additionally, 642 OTUs were shared between the rumen and small intestines, and 545 OTUs were shared between the small and large intestines. Analysis revealed 558 specific OTUs in the rumen, 234 OTUs in the small intestine, and 665 OTUs in the large intestine. Notably, the small intestine exhibited significantly fewer unique bacterial OTUs than either the rumen or the large intestine, reflecting its transitional role between the foregut and hindgut microbial communities.

Alpha diversity analysis of luminal contents collected from the rumen, small intestine, and large intestine indicated sequencing coverage ≥ 0.99 for all samples (Figure 1b and Supplementary Figure S2), confirming that the sequencing depth was sufficient in comprehensively capturing the microbial communities. The analysis demonstrated that bacterial diversity, richness, and evenness were highest in the large intestine (colon) and lowest in the small intestine.

The NMDS ordination plots (Figure 1c,d) reveal clear clustering patterns corresponding to the rumen, small intestine, and large intestine samples. The sharp segregation of these clusters highlights marked spatial heterogeneity in microbial community composition across the GI tract of Qianqiu goats.

### 2.2. Microbial Profiles of Three GI Segments

**Figure 2:**
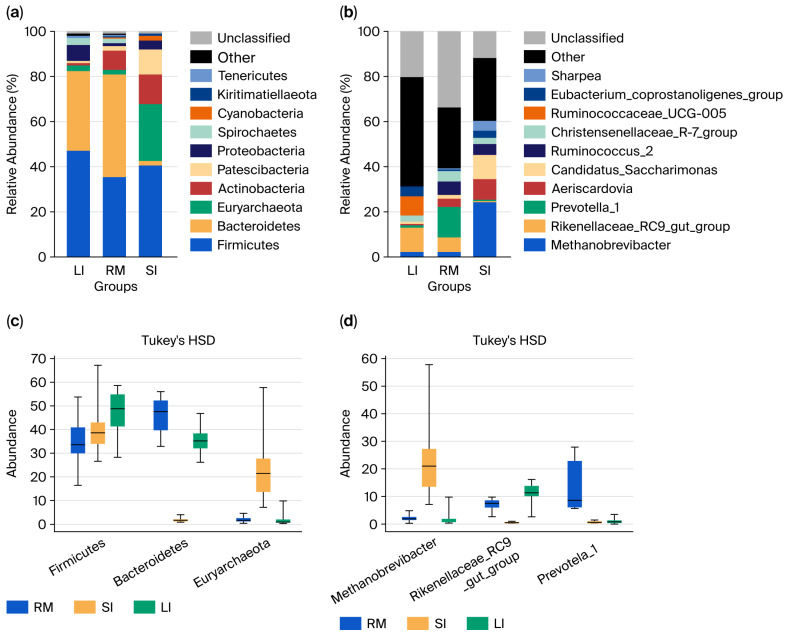
Microbial composition of the three gastrointestinal segments: rumen (RM), small intestine (SI), and large intestine (LI). (**a**) Phylum-level microbial composition in each segment. (**b**) Genus-level microbial composition in each segment. (**c**) Differentially abundant taxa at the phylum level between different segments. (**d**) Differentially abundant taxa at the genus level between different segments.

At the phylum level (Figure 2a), *Firmicutes* dominated across all samples. In the rumen, *Firmicutes*, *Bacteroidota*, and *Actinobacteria* accounted for ~88% of the community, whereas the small intestine was characterized by *Firmicutes*, *Euryarchaeota*, and *Actinobacteria* (78.57%). The large intestine was dominated by *Firmicutes*, *Bacteroidota*, and *Proteobacteria* (89%). Along the GI tract, *Firmicutes* and *Proteobacteria* increased from the rumen to the large intestine; *Bacteroidota* decreased, then increased; and *Actinobacteria* peaked in the small intestine before declining. *Euryarchaeota* were specifically enriched in the small intestine. Statistical analysis revealed significant differences in the distribution of *Bacteroidota* and *Euryarchaeota* among the three regions. *Actinobacteria* were significantly higher in the rumen and small intestine than in the large intestine (*p* < 0.05).

At the genus level (Figure 2b), the rumen was dominated by *Rikenellaceae_RC9_gut_group*, *Prevotella_1*, *Ruminococcus_2*, and *Christensenellaceae_R-7_group*. The small intestine exhibited high abundances of *Methanobrevibacter*, *Rikenellaceae_RC9_gut_group*, *Candidatus Saccharimonas*, and *Aeriscardovia*, while the large intestine was enriched in the *Rikenellaceae_RC9_gut_group*, *Ruminococcaceae_UCG_005*, *Eubacterium coprostanoligenes_group*, and *Christensenellaceae_R-7_group*. Spatial patterns revealed that *Methanobrevibacter* and *Candidatus Saccharimonas* peaked in the small intestine; *Prevotella_1*, *Ruminococcus_2*, and *Christensenellaceae_R-7_group* were highest in the rumen; and *Ruminococcaceae_UCG_005* and *Eubacterium coprostanoligenes_group* increased along the GI tract. Statistical analysis (Figure 2c,d) confirmed significant differences in the distribution of *Methanobrevibacter*, *Rikenellaceae_RC9_gut_group*, *Prevotella_1*, *Aeriscardovia*, and *Candidatus Saccharimonas among the three GI regions* (Kruskal–Wallis raw *p* < 0.05; Tukey HSD raw *p* < 0.05), reflecting region-specific microbial assemblages shaped by distinct physicochemical environments along the gastrointestinal tract of Qianqiu goats.

### 2.3. Analysis of Differential Genera in the GI

**Figure 3:**
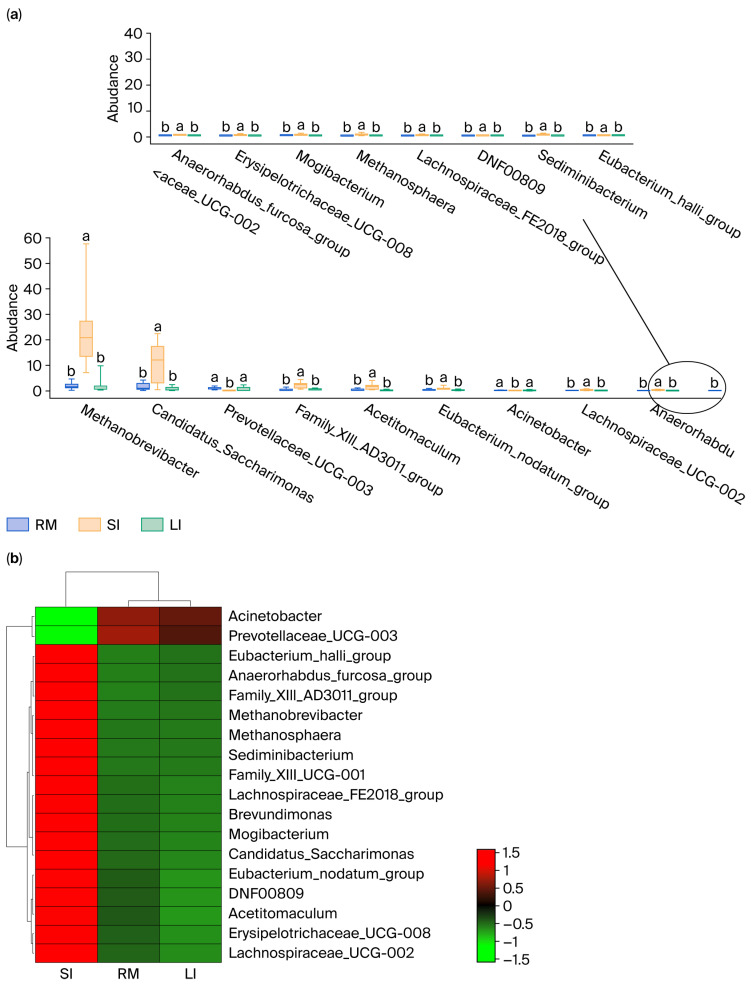
(**a**) Tukey’s HSD analysis of key genera abundance; (**b**) heatmap of key genera abundance. Different lowercase letters above the boxplots indicate significant differences among treatments according to Tukey’s HSD test (*p* < 0.05), and the circlein panel (**a**) is only used to show that the 18 differential genera are split into two coordinate axes because they could not be clearly displayed in a single axis; it has no additional statistical meaning.

An in-depth analysis was conducted for the differential microbiota in the rumen, small intestine, and large intestine of Qianqiu goats, focusing on genera that exhibited significant differences in abundance between the rumen and small intestine, as well as between the large intestine and small intestine (*p* < 0.05). By selecting microbial taxa that met this criterion, 18 key genera were ultimately identified (Figure 3a; Supplementary Table S1), and a heatmap was generated to show their relative abundance in the rumen, small intestine, and large intestine (Figure 3b).

*Methanobrevibacter* had a relative abundance of 1.92% in the rumen, which was significantly higher at 24.51% in the small intestine, showing a notable shift in abundance. Similarly, *Candidatus_Saccharimonas* had a relative abundance of 1.63% in the rumen, which increased to 10.85% in the small intestine, displaying a significant difference. In addition, genera, such as *Prevotellaceae_UCG-003*, *Family_XIII_AD3011_group*, *Methanosphaera*, and *Acetitomaculum* also exhibited significant differences in abundance between the rumen and small intestine. For example, *Prevotellaceae_UCG-003* had a relative abundance of 0.95% in the rumen, but this was only 0.03% in the small intestine. Similarly, genera such as *Candidatus_Saccharimonas* and *Prevotellaceae_UCG-003* also showed significant differences in abundance between the large and small intestines. *Candidatus_Saccharimonas* had a relative abundance of 1.63% in the large intestine and 10.85% in the small intestine, while *Prevotellaceae_UCG-003* had a relative abundance of 0.95% in the large intestine, but its value dramatically dropped to 0.03% in the small intestine. Moreover, other genera, such as *Family_XIII_AD3011_group* and *Acetitomaculum*, displayed similar patterns of varying abundance, indicating a clear difference in their distribution between the large and small intestines. For instance, *Family_XIII_AD3011_group* had a relative abundance of 0.45% in the large intestine; conversely, in the small intestine, this value was 2.04%, showing a marked difference.

Overall, the relative abundance of several genera in the small intestine (SI) was significantly higher than in both the rumen (RM) and large intestine (LI). Additionally, certain genera, such as *Prevotellaceae_UCG-003*, which were relatively abundant in the rumen and large intestine, exhibited lower abundance in the small intestine, potentially reflecting their primary role in fiber degradation and fermentation processes [24].

### 2.4. Differential Metabolites in the GI Tract of Qianqiu Goats

**Figure 4:**
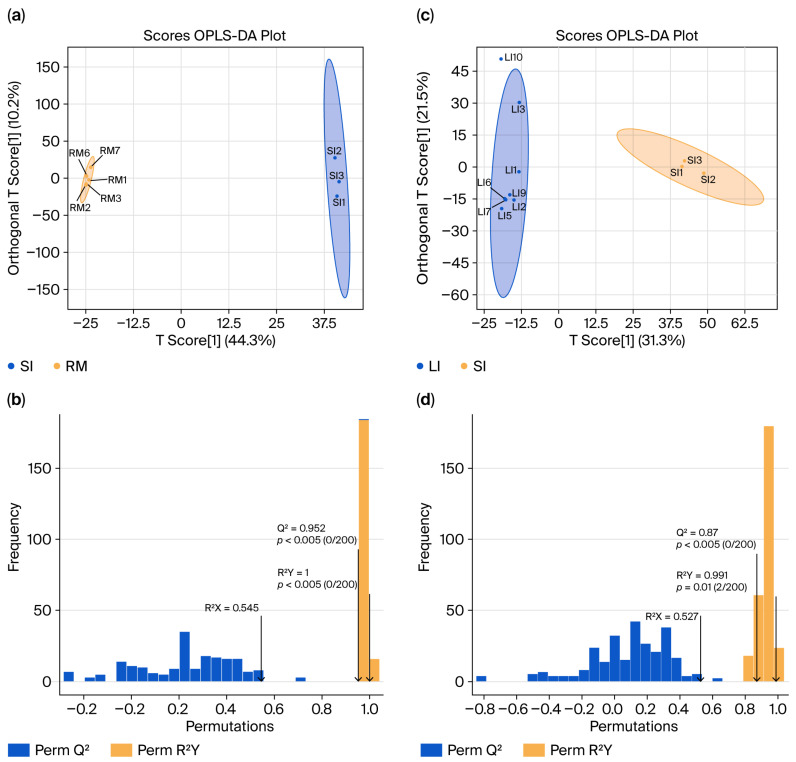
Orthogonal Partial Least Squares (OPLS) score plots and model validation results. (**a**) “SI vs. RM” score plot showing sample separation; (**b**) permutation/cross-validation results for the “SI vs. RM” model. (**c**) “LI vs. SI” score plot showing sample separation; (**d**) permutation/cross-validation results for the “LI vs. SI” model.

To investigate the metabolic profiling characteristics of the GI tract in Qianqiu goats, we conducted non-targeted metabolomics using ultra-high-performance liquid chromatography–mass spectrometry on samples from the rumen, small intestine, and large intestine. The resulting Orthogonal Partial Least Squares Discriminant Analysis (OPLS-DA) model—which separates variation predictive of sample class from orthogonal (non-class-related) variation—was used to generate score plots (Figure 4a,c) and validation plots (Figure 4b,d). The analysis revealed significant differences in metabolite composition between the SI and RM groups (SI vs. RM) and the LI and SI groups (LI vs. SI), with clear group separations. Permutation tests confirmed the robustness of the models, which demonstrates excellent predictive ability and fit without the risk of overfitting.

### 2.5. Data Quality Control, Soft-Threshold Selection, and WGCNA Module Construction

**Figure 5:**
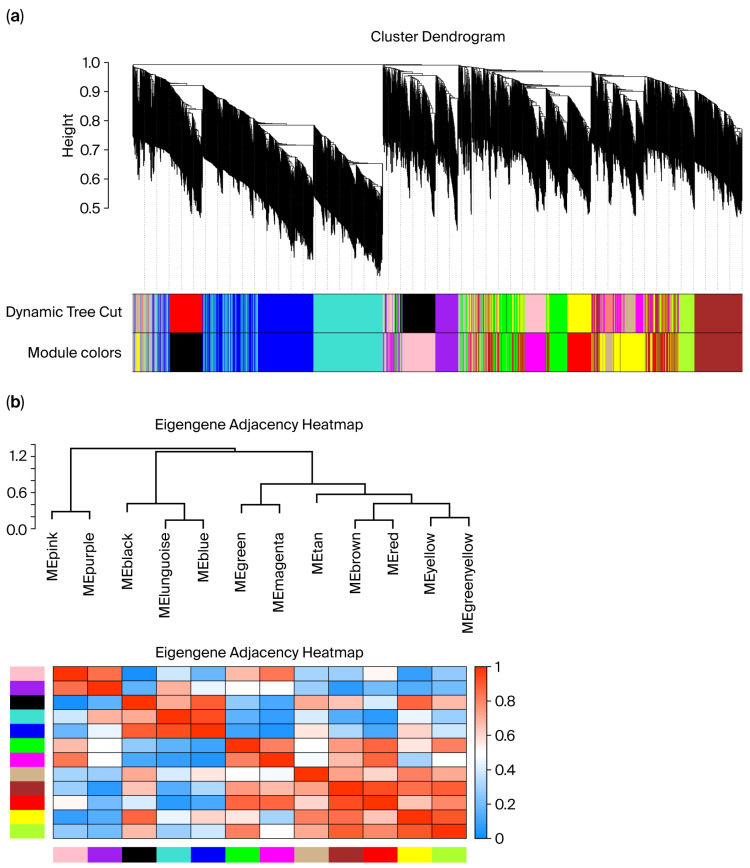
(**a**) Sample clustering relationship diagram; (**b**) module generation plot. In panel (**a**), the different colors under the dendrogram represent distinct co-expression modules identified by WGCNA. In panel (**b**), the color scale from blue to red indicates the eigengene adjacency (correlation) between modules, with blue representing low adjacency and red representing high adjacency.

During data quality control, violin plots of metabolite intensities showed approximately symmetric, bell-shaped distributions for each sample with no aggregation of extreme outliers. This indicated that log transformation and normalization yielded comparable data across tissues (Supplementary Figure S3). Sample-level hierarchical clustering grouped all profiles strictly by tissue type, with no clear outliers identified (Figure 5a). Network construction used the topological overlap matrix (TOM) to quantify metabolite–metabolite connectedness and the dynamic tree cut algorithm to define modules; the dendrogram (top) displays metabolite clustering, and the color bar (bottom) denotes the module membership (Figure 5a). Using this procedure, several co-expression modules were identified (e.g., blue, turquoise, and brown), each representing metabolites with highly correlated expression patterns within modules and comparatively lower correlation between modules (Figure 5b). These modules provide the basis for downstream analyses of tissue-associated metabolic pathways and biomarker prioritization.

### 2.6. Correlation and Expression Profiles of Tissue-Associated Modules

**Figure 6:**
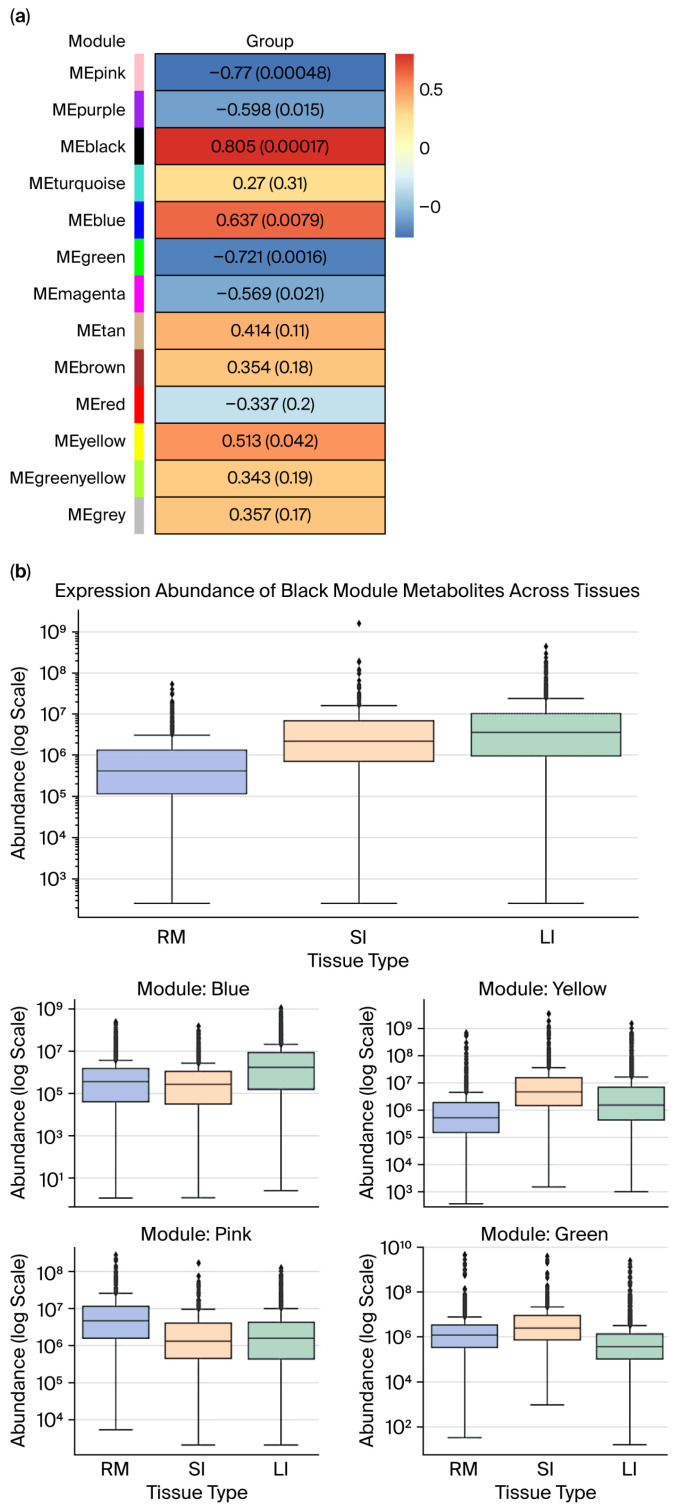
(**a**) Module–group correlation *p*-value plot (rows represent module eigengene (ME), and columns represent “group” for each intestinal segment: RM, SI, and LI), where each cell shows the correlation and *p*-value, (the table is color-coded by correlation). (**b**) Expression abundance of metabolite modules across gastrointestinal tissues (RM, SI, and LI) in Qianqiu goats. (Box plots show the distribution of metabolite abundances across the three intestinal segments (RM, SI, LI) for modules that are significantly associated with tissue type). In panel (**a**), the colors on the left indicate the different WGCNA modules (MEpink, MEpurple, MEblack, etc.), and the color scale in the heatmap (from blue to red) shows the strength and direction of the correlation between each module and group (blue = negative, red = positive). In panel (**b**), the colors of the box plots correspond to the different tissue types as indicated in the legend, and the dots represent the individual sample values (outliers) within each group.

To examine tissue-specific metabolite patterns, WGCNA was employed to correlate MEs with tissue types numerically coded as follows: rumen = 0; small intestine = 1; and large intestine = 2. Seven metabolite modules showed significant associations (*p* < 0.05; Figure 6a). Modules with strong ME–trait correlations and internal coherence (a significant gene significance–module membership (GS–MM) correlation, Supplementary Figures S4–S8) were further analyzed across tissues (Figure 6b).

The black module displayed the strongest positive correlation (r = 0.805, *p* < 0.05) and high internal consistency (GS–MM r = 0.59, *p* < 0.05). Boxplot analysis revealed significantly higher expressions in the small and large intestines compared to the rumen, with the small intestine showing the highest median. Both gut regions exhibited broad expression ranges and high-abundance outliers, which are hallmarks of hub metabolites with tissue-specific regulation.

The blue module was positively associated with distal gut tissues (r = 0.637, *p* < 0.05; GS–MM r = 0.52, *p* < 0.05), and its expression peaked in the large intestine, showing elevated medians and upper quartiles as well as extreme outliers. This highlights its functional relevance to microbial fermentation, bile acid metabolism, or ion balance in the colon.

The yellow module, although marginally correlated (r = 0.513, *p* < 0.05), demonstrated robust GS–MM alignment (r = 0.52, *p* < 0.05). Expression was highest in the small intestine, with considerable variability, suggesting small intestine-specific metabolic activity and potential as a biomarker ensemble.

In contrast, the pink and green modules were negatively correlated with tissue type (pink: r = −0.77; *p* < 0.05; GS–MM r = −0.56; *p* < 0.05; green: r = −0.721; *p* < 0.05; GS–MM r = −0.47; *p* < 0.05), indicating repressed expression in gut tissues. Expression of the pink module was highest in the rumen; however, it remained consistently low with minimal variance in the small and large intestines. This finding implies the existence of rumen-specific metabolic roles. The green module showed comparable expression in the rumen and small intestine but lower levels in the large intestine, suggesting that metabolic processes are specialized to regions and dependent on gut segments rather than subject to a uniform absorptive function.

### 2.7. KEGG Pathway Enrichment and Hub-Metabolite Network Analysis

**Figure 7:**
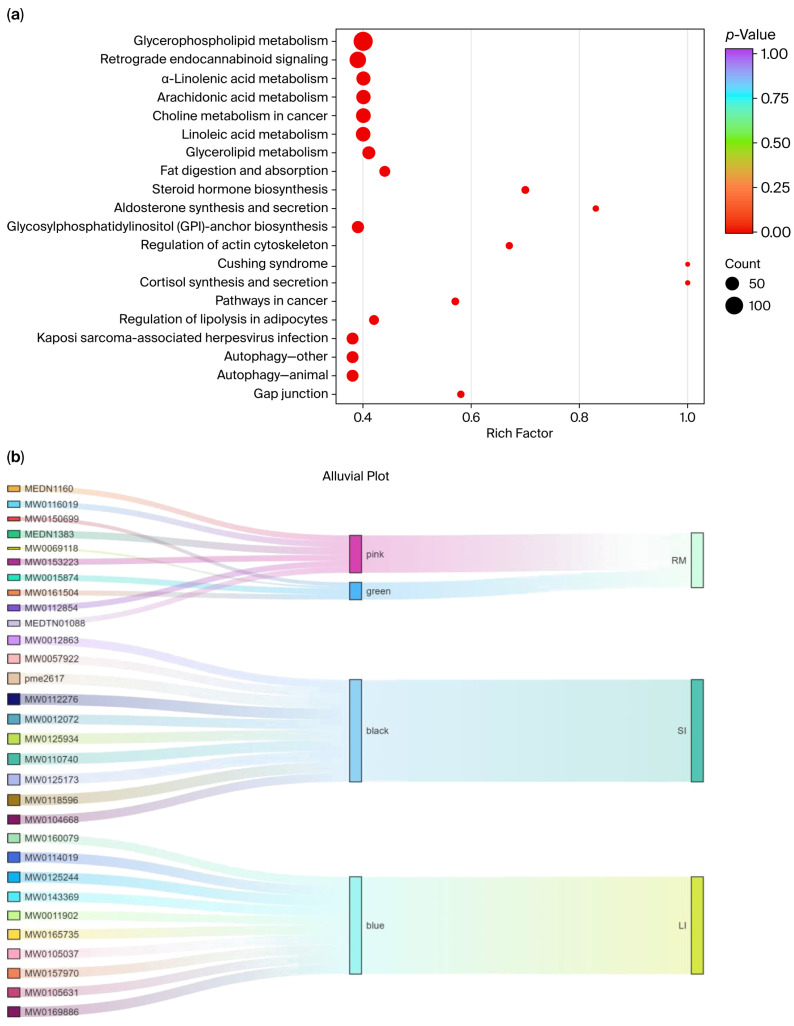
(**a**) KEGG pathway enrichment analysis; (**b**) Sankey visualization of the three-layer tissue–module–metabolite structure. Each ribbon links an individual metabolite (**left**, labeled by Metabolite_ID) to its WGCNA module (**middle**, colored “pink/green/black/blue”) and to associated intestinal tissue (**right**, RM; SI; LI).

Kyoto Encyclopedia of Genes and Genomes (KEGG) enrichment analysis was performed on five tissue-associated modules (black, blue, yellow, pink, and green), and the pathways enriched within each module were summarized (Figure 7a). The top 20 KEGG enrichment results are listed in Supplementary Table S2. Most significantly enriched pathways fell within the metabolism category (88.81%). Among them, glycerophospholipid metabolism (49.46%), arachidonic acid metabolism (23.10%), linoleic acid metabolism (22.74%), and choline metabolism (22.74%) were the most prominent, underscoring the central roles of lipid signaling and membrane-lipid remodeling in metabolic regulation. In addition, enrichment was observed for steroid hormone biosynthesis, fat digestion and absorption, and glycosphingolipid metabolism, further supporting links to lipid-energy metabolism and hormone-signaling regulation.

Beyond metabolism, several disease-related and environmental information-processing pathways were also represented, including insulin resistance, choline metabolism in cancer, Cushing syndrome, and the PI3K–Akt and AMPK signaling pathways. Notably, glycerophospholipid metabolism, arachidonic acid metabolism, linoleic acid metabolism, and retrograde endocannabinoid signaling showed strong enrichment in KEGG analysis (*p* < 0.01), indicating that a high proportion of the metabolites detected were mapped to these pathways relative to the background metabolite set.

We ranked metabolites in each module based on GS and visualized the tissue–module–metabolite relationships using Sankey diagrams (Supplementary Figure S9 and Figure 7b). In the rumen, core metabolites (Supplementary Table S3) from the pink and green modules included mevalonate-5-phosphate and Triglyceride (TG) (15:0/16:1/18:4). In the small intestine, core metabolites (Supplementary Table S4) from the black module included PC 14:0/18:0 and L-methionine sulfoxide. In the large intestine, core metabolites (Supplementary Table S5) from the blue module included anserine and sulfamonomethoxine.

### 2.8. Integrated Microbe–Metabolite Network of Qianqiu Goats

**Figure 8:**
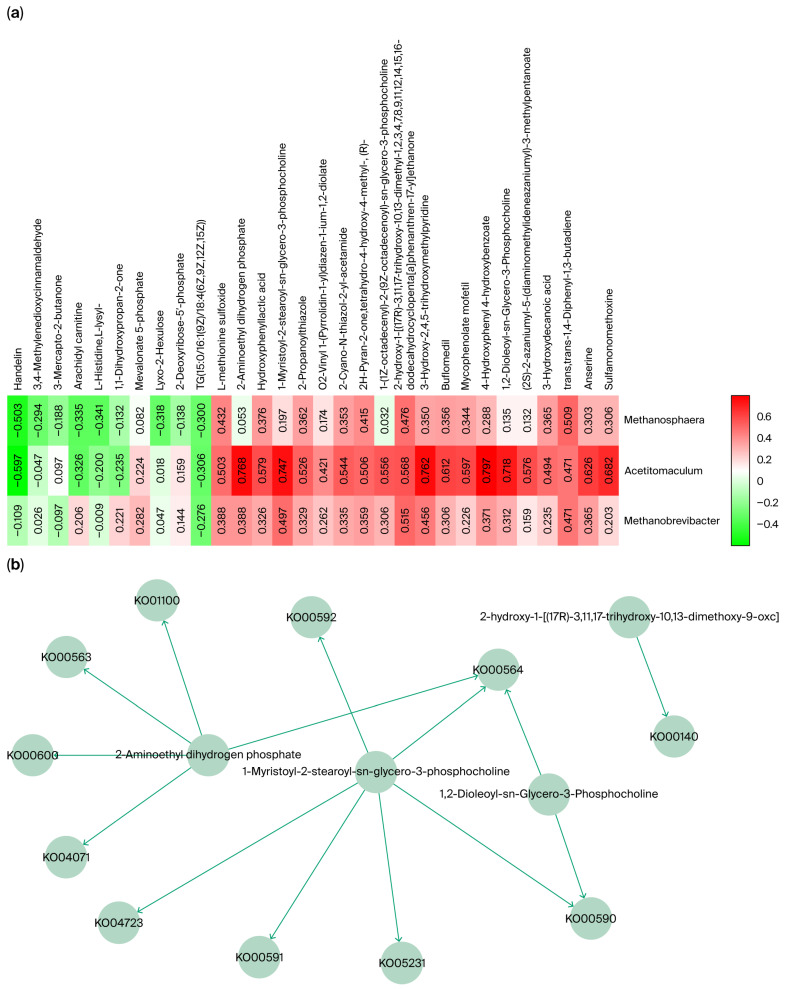
(**a**) Spearman correlation analysis of key metabolites and microbial abundance. (**b**) Metabolic pathway network of key metabolites associated with microbial abundance.

Eighteen differential genera were further analyzed, and the relative abundances of *Methanobrevibacter*, *Acetitomaculum*, and *Methanosphaera* were significantly higher in the small intestine than in other GI segments of Qianqiu goats. This distribution pattern has been rarely observed in species [25,26]. To explore the potential role of these three genera in the differential abundance in the small intestine, a Spearman correlation analysis (a non-parametric method used to assess the strength and direction of association between two ranked variables) was conducted between the identified core metabolites and the genera (Figure 8a). The results showed that these genera were significantly correlated with several metabolites, which were enriched in specific metabolic pathways (Figure 8b).

For *Methanobrevibacter*, the significantly correlated metabolite, 2-hydroxy-1-[(17R)-3,11,17-trihydroxy-10,13-dimethoxy-9-oxohentriacontyl], was enriched in the Ko00140 (a specific KEGG orthology (KO) identifier) steroid biosynthesis pathway. This is consistent with *Methanobrevibacter*’s documented capacity to tolerate bile-rich niches via membrane stabilization [27]. *Acetitomaculum* was significantly and positively correlated with metabolites involved in several lipid metabolism pathways, including Ko00590, Ko00591, Ko00592, Ko00564, and Ko04071, indicating its active involvement in intestinal lipid turnover [28]. For *Methanosphaera*, the significantly correlated metabolite was trans, trans-1,4-Diphenyl-1,3-butadiene, which was not enriched in any specified pathways.

## 3. Discussion

Recent advances in high-throughput sequencing and metagenomics have rapidly expanded research on ruminant GIT microbiota beyond the rumen [29,30]. Studies now cover multiple compartments along the entire digestive tract, including both the small and large intestines. For example, a recent large-scale study reconstructed a Goat Multi-Kingdom Microbiome Catalog (GMMC), based on 497 GIT samples from ten anatomical sites ranging from the rumen to the colon [31]. The catalog comprises 4004 bacterial MAGs, 71 archaeal MAGs, and 7204 viral genomes. This study is a comprehensive, genome-level resource covering bacteria, archaea, and viruses across the gastrointestinal tract of goats. Importantly, this study demonstrates that archaea are not confined to the rumen or large intestine; they are detectable throughout the GIT, including the small intestine (SI). In addition, previous metagenomic studies that sampled different developmental stages (e.g., goat kids) and various gut compartments (e.g., stomach, small intestine, and large intestine) have also revealed compartment-specific microbial community assembly and compositional differences along the GIT [32].

On this basis, we conducted a systematic analysis of the rumen, small intestine (SI), and large intestine (LI) of the Qianqiu goat. The results revealed marked differences in microbial community structure and composition among the three gut segments. These findings are consistent with the spatial heterogeneity along the gastrointestinal tract documented in studies such as the Goat Multi-Kingdom Microbiome Catalog (GMMC) [31]. Notably, in the small intestine (SI), pronounced enrichment of certain archaeal genera was observed, particularly Methanobrevibacter and Methanosphaera, with relative abundances up to 24.51% and 0.43%, respectively. Concurrently, bacterial genera associated with lipid and acetate metabolism, such as Acetitomaculum [25], also exhibited relatively high abundance in the SI (~1.58%).

Although previous studies have encompassed multiple GIT compartments (including the small intestine, SI), virtually no systematic, quantitative reports (e.g., relative-abundance or enrichment analyses) have been published on methanogens in the small intestine of ruminants, particularly goats or young lambs [33,34]. In prior work, even when intestinal samples (e.g., jejunum and ileum) were collected, analyses typically relied on 16S rRNA metabarcoding. Such research only documented the presence or taxonomic composition of archaea, rather than applying MAG reconstruction combined with abundance estimation to reveal archaeal enrichment in the SI [35]. The present data indicate that the small intestine may constitute a key habitat and active zone for archaea (especially certain genera). Meanwhile, the observed enrichment of bacterial populations associated with lipid and acetate metabolism within the SI suggests that this region may play a significant but previously under-appreciated role in host–microbe interactions underlying energy metabolism [36,37].

To further investigate the potential biological functions of the specific taxa enriched in the small intestine (SI), a Spearman correlation analysis was conducted between their relative abundances and the core metabolites identified in the SI. The analysis revealed that the abundance of Methanobrevibacter correlated positively and significantly with several lipid-related metabolites (*p* < 0.05), suggesting that these archaea may participate in or influence the lipid metabolic environment of the SI. This observation was consistent because a bile salt hydrolase gene (bsh) was identified in Methanobrevibacter smithii [38]. The presence of this gene has been shown to confer enhanced survival under bile stress from deconjugation of bile salts [39]. Furthermore, previous studies have provided evidence that bile acids and steroids can be transformed by microorganisms to confer ecological advantages in bile-rich environments [40]. On this basis, it is hypothesized that bile tolerance in Methanobrevibacter in the small intestine of Qianqiu goats may be enhanced by modulating lipid and steroid metabolism, thereby facilitating persistence and functional activity in this compartment.

It should be noted, however, that although BSH homologs have been widely reported across gut microbial communities [41], their distribution appears to be largely confined to bacteria [42]. Direct functional evidence of BSH activity in archaea is absent in most studies [43]. This caveat warrants caution when extrapolating bacterial-based mechanisms to archaeal taxa such as Methanobrevibacter.

Moreover, recent work has expanded the recognized repertoire of BSH functions. In addition to classical bile salt deconjugation, BSH (or BSH-like enzymes) has been shown to mediate amine N-acyltransferase activity, leading to the formation of microbial-conjugated bile acids (MCBAs), which may modulate bile acid pool composition, microbial colonization, lipid absorption, and host–microbe signaling [44,45,46]. Therefore, while the positive correlation between Methanobrevibacter abundance and lipid-related metabolites in the SI provides a plausible indication of lipid/steroid metabolic modulation and potential bile tolerance, functional validation, including demonstration of BSH (or analogous enzyme) expression in SI Methanobrevibacter, measurement of bile salt transformation products, and assessment of membrane lipid composition, remains necessary.

Similarly, the abundance of Acetitomaculum showed strong positive correlations with multiple metabolites associated with glycerophospholipid metabolism, ether-lipid metabolism, and other lipid metabolic pathways (*p* < 0.05). This correlation suggested that Acetitomaculum, and by extension, reductive acetogenic bacteria, may have been involved not only in acetate production [47]. Given that acetate can serve as a carbon and energy precursor (via acetyl-CoA) for lipid biosynthesis, it was hypothesized that the acetate they release might also be utilized by microbes (or the host) in the intestinal milieu for membrane lipid synthesis or remodeling [47]. This could influence microbial community structure and energy metabolism in the gut; however, direct experimental evidence is lacking. This interpretation was further supported by a recent genome-resolved metagenomic survey, which revealed that reductive acetogenic bacteria were highly enriched in the hindgut (cecum) of ruminants; in that study, acetogens were approximately 12 times more abundant in the cecum than the rumen [48]. In situ measurements in the same work showed that dissolved hydrogen and acetate levels were significantly higher in the cecum than in the rumen, consistent with enhanced reductive acetogenesis and diminished methanogenesis in the hindgut environment [48].

Based on these observations, it is reasonable to propose that Acetitomaculum (or related acetogens) is important as a “carbon/energy provider” in the hindgut: acetate produced by reductive acetogenesis might have been used as a carbon backbone or acyl-group source for membrane lipid synthesis or remodeling [47], thus contributing to the generation of metabolite signatures associated with glycerophospholipid, ether-lipid, and other lipid metabolic pathways observed in our metabolomic analysis.

Methanogens and bacteria associated with lipid/acetate metabolism were concurrently enriched in the small intestine (SI) of the Qianqiu goat. The classical view is that methanogenesis in ruminants (especially in the rumen) is driven by methanogens utilizing H_2_ + CO_2_ (or formate), which function as an H_2_/electron sink to maintain fermentation balance. Thus, a hypothetical mechanism is proposed [49]. If active methanogenesis is maintained in the SI, H_2_ and electrons produced by other microbes can be consumed, resulting in low H_2_ partial pressure and a favorable redox balance, thus providing stable conditions for other anaerobic processes (e.g., fermentation, lipid metabolism, and intermediate metabolite production) [49]. Concurrently, under such stable conditions, lipid/acetate-metabolizing bacteria (e.g., Acetitomaculum) can utilize dietary components (or host secretions), fermentation substrates, or metabolic by-products to generate acetate or lipid/membrane-lipid intermediates [50]; these intermediates can be subsequently absorbed by the host intestinal epithelium for membrane lipid synthesis/remodeling or energy/lipid metabolism [51]. This can potentially influence epithelial membrane integrity, nutrient/lipid uptake capacity, and host energy/lipid metabolic homeostasis [52]. If this hypothesis is confirmed, the small intestine (SI) of the Qianqiu goat may be regarded not solely as a transit/absorption site, but as a distinct microbial niche and metabolic compartment: a “microbial–host metabolic compartment”.

However, it is important to acknowledge several limitations of this study. Although correlation analysis was employed, it cannot establish causality, and other factors, such as the host’s physiological state, secretory activity, or environmental conditions, may have contributed to the microbial and metabolic changes observed [53]. Additionally, our analysis was based on intestinal lumen contents rather than mucosa-associated microbiota; this may have excluded some important microbial interactions at the epithelial surface [54,55]. Furthermore, direct functional validation, such as measurements of methane production or metabolic enzyme activities, was not performed; thus, our conclusions were only based on correlations [56]. Finally, as this study focused solely on Qianqiu goats, the generalizability of these findings to other ruminant species or varying farming conditions remains unclear.

To address these limitations, future research should validate microbiota distribution across different gut regions [57] using metagenomics, transcriptomics, and functional analyses to elucidate specific microbial activities [58]. Additionally, further studies should assess the generalizability of this model to other ruminants and farming conditions to evaluate its broader applicability [59]. Exploring the underlying mechanisms of microbial–metabolite–host interactions and their ecological roles will help expand our understanding of gut microbiota dynamics and their implications for animal health and productivity.

In conclusion, this study introduces a new conceptual framework for understanding the spatial heterogeneity of microbiota and metabolites in the gastrointestinal tract of Qianqiu goats, particularly within the small intestine. The proposed “small intestine–methanogen/lipid/bile-adaptive micro-ecosystem” model not only challenges conventional views of ruminant gut ecology but also provides new avenues for exploring microbiota–metabolite–host interactions and optimizing feeding and nutritional strategies. While functional validation and mechanistic studies are necessary, our findings establish a foundation for future research and offer significant theoretical and practical implications for ruminant farming and feed optimization.

## 4. Materials and Methods

A total of 24 goats were collected from 6-month-old Qianqiu goats (Anhui Tianchang Zhou Sheep Industry Co., Ltd., Tianchang, China) (BW: 28.40 ± 1.80 kg), which were equally divided into three groups: the rumen (RM), small intestine (SI), and large intestine (LI). All animal experiments were approved by the Science and Technology Ethics Committee of Anhui Agricultural University (Protocol number: KJLL2024032).

The instruments used for sample collection included a 10 mL syringe barrel (needle-removed), 5 mL sterile sample tubes, dry ice, surgical tools, and other necessary equipment. To collect luminal contents, the barrel was carefully inserted into the rumen and the small (mid-jejunum) and large intestines (sigmoid colon), ensuring full immersion in luminal contents. The target intestinal segment was gently massaged to facilitate extraction, and a mixture of luminal contents and fluids was drawn into the syringe. The sample was transferred into labeled sterile tubes, placed temporarily on dry ice, and then processed within 30 min: parallel aliquots were stored at –20 °C and –80 °C for short-term preservation, while some were immediately immersed in liquid nitrogen for long-term storage. The exposed intestinal segment was then incised; the surrounding tissue was carefully excised, placed in sterile tubes, labeled, and stored identically.

For tissue collection, samples of gastrointestinal segments were obtained from six-month-old Qianqiu goats during slaughter. Rumen content was collected by opening the rumen directly; samples from the small and large intestines were collected via longitudinal incision, and contents were either collected directly or rinsed with saline as required. All specimens were immediately stored at approximately –80 °C to preserve integrity for subsequent analyses. Gastrointestinal tissue samples were rinsed with sterile physiological saline, transferred to labeled sterile tubes, stored at –80 °C for 24 h, and then moved to liquid nitrogen for long-term storage. All handling was performed under aseptic conditions to prevent contamination and ensure the integrity of samples used for subsequent metabolomic studies.

Genomic DNA was extracted using the HiPure Stool DNA Kit (D3141, Megagen, Guangzhou, China) following the manufacturer’s instructions. The amplification steps are referenced in the literature [34].

The relative abundance of bacterial taxa was visualized using Krona [35], bar plots (ggplot2 [36]), chord plots (circlize [37]), and heatmaps (pheatmap [38]). Correlation analyses were conducted using the psych package (R package 1.0.13) [39]. Alpha diversity indices, including ACE, Chao1, and Shannon, were calculated using QIIME (v1.7.0), and non-metric multidimensional scaling (NMDS) was employed to evaluate dissimilarity in microbial community structures among the three GI segments.

For metabolomics, samples stored at −80 °C were thawed on ice and ground in liquid nitrogen. A 400 μL solution (methanol/water = 7:3, *V/V*) containing an internal standard was added to a 20 mg ground sample, and shaken at 1500 rpm for 5 min. After being placed on ice for 15 min, the sample was centrifuged at 12,000 rpm for 10 min (4 °C). In total, 300 μL of the supernatant was collected and placed in −20 °C conditions for 30 min. The sample was then centrifuged at 12,000 rpm for 3 min (4 °C). A total of 200 μL aliquots of supernatant were transferred for LC-MS analysis.

All samples were analyzed under positive ion conditions and eluted from the T3 column (Waters ACQUITY Premier HSS T3 Column 1.8 µm, 2.1 mm × 100 mm). We used 0.1% formic acid in water as solvent A and 0.1% formic acid in acetonitrile as solvent B for the following gradient: 5 to 20% for 2 min, increased to 60% for the following 3 min, before increasing to 99% for 1 min and holding for 1.5 min. Then, we returned to 5% mobile phase B for 0.1 min, which was held for 2.4 min. The analytical conditions were as follows: column temperature, 40 °C; flow rate, 0.4 mL/min; and injection volume, 4 μL. The methods alternated between full-scan MS and data-dependent MSn scans using dynamic exclusion. MS analyses were carried out with electrospray ionization in the positive ion mode and negative ion mode using full-scan analysis over *m/z* 75–1000 at 35,000 resolution. Additional MS settings are listed as follows: ion spray voltage, 3.5 KV or 3.2 KV in positive or negative modes, respectively; sheath gas (Arb), 30; aux gas, 5; ion-transfer tube temperature, 320 °C; vaporizer temperature, 300 °C; collision energy, 30, 40, 50 V; signal intensity threshold, 10^6^ cps; top N vs. Top speed, 10; and exclusion duration, 3 s.

Data were log2-transformed and analyzed in R (version 4.5.2; R Foundation). Principal component analysis (PCA), hierarchical cluster analysis, and OPLS-DA (VIP > 1, *p* < 0.05) were performed with model validation via permutation testing, using the ropls package (version 1.42.0; Bioconductor 3.22). Heatmaps were generated with pheatmap (version 1.0.13). Metabolites were annotated and analyzed for pathway enrichment using the KEGG database.

To identify co-expressed metabolite clusters, WGCNA was used to construct an exploratory scale-free metabolite network (*n* = 10, below recommended size). Modules were identified with TOM and dynamic tree cutting (minModuleSize = 8 and mergeCutHeight = 0.25). Module eigengenes correlated with sample traits using Pearson r and raw *p*, implemented via the WGCNA R package (version 1.73). KEGG enrichment raw *p*-values were computed and used solely to rank pathways. Metabolite–metabolite correlations (|r| ≥ 0.7) with raw *p* were retained for candidate prioritization.

All statistical analyses were conducted in R (v4.5.2). Alpha-diversity indices were compared among intestinal segments using one-way ANOVA followed by Tukey’s HSD post hoc test; assumptions of normality and homogeneity of variance were verified using Shapiro–Wilk and Levene’s tests, respectively, and data were log-transformed when necessary. Microbial-community dissimilarities (NMDSs) were assessed using Bray–Curtis distances and tested with PERMANOVA (999 permutations). Metabolomics data (log_2_-transformed) were analyzed via PCA and hierarchical cluster analysis; OPLS-DA models were validated using permutation testing (200 permutations); and variables satisfying VIP > 1 and *p* < 0.05 were considered significant. Multiple-testing corrections for module–group correlations were performed by controlling the false discovery rate (FDR < 0.05). All tests were two-tailed, and significance was set at α = 0.05.

## 5. Conclusions

Across the rumen and small and large intestines of Qianqiu goats, amplicon sequencing revealed a highly diverse and evenly distributed microbiota with strong functional redundancy: Prevotella dominated the rumen; Methanobrevibacter and Candidatus Saccharimonas dominated in the small intestine; and Rikenellaceae_RC9_gut_group plus Bacteroides were most significant in the large intestine. The unexpected enrichment of Methanobrevibacter and the near-absence of Prevotellaceae_UCG-003 in the small intestine indicate a unique, bile-tolerant anaerobic niche in this segment. Metabolome-wide WGCNA further identified 13 segment-specific metabolite modules; small-intestinal hubs such as 1-Myristoyl-2-stearoyl-sn-glycero-3-phosphocholine, L-methionine sulfoxide, and 2-Aminoethyl dihydrogen phosphate assemble an absorption–antioxidant–steroid metabolic network.

Integrative correlation analysis indicates that Methanobrevibacter, Methanosphaera, and Acetitomaculum of the small intestine co-occur in a hydrogen-driven syntrophic module: methanogens likely scavenge H_2_ produced by Acetitomaculum, which may reduce CO_2_ to acetate. Furthermore, their associations with steroid/lipid-related pathways point to possible membrane-remodeling adaptations in a bile-rich lumen. Therefore, these associations suggest a host-specific strategy in Qianqiu goats that may optimize hydrogen turnover, SCFA supply, and intestinal homeostasis, conferring a competitive advantage. However, causal verification of this hypothesis remains to be established.

## Data Availability

The original contributions presented in this study are included in the article/Supplementary Material. Further inquiries can be directed to the corresponding author.

## Supplementary Materials

The following supporting information can be downloaded at https://www.mdpi.com/article/10.3390/ijms262411815/s1.
