# Supplementary material for: Spatial Multi-Omics Analysis of the Qianqiu Goat Gut Microbiome and Metabolome
Source: Int J Mol Sci. 2025 Dec 7;26(24):11815. doi: 10.3390/ijms262411815 (PMC12732871; doi:10.3390/ijms262411815)
Supplement: Supplementary file 1 [file ijms-26-11815-s001.zip › Supplementary.pdf]

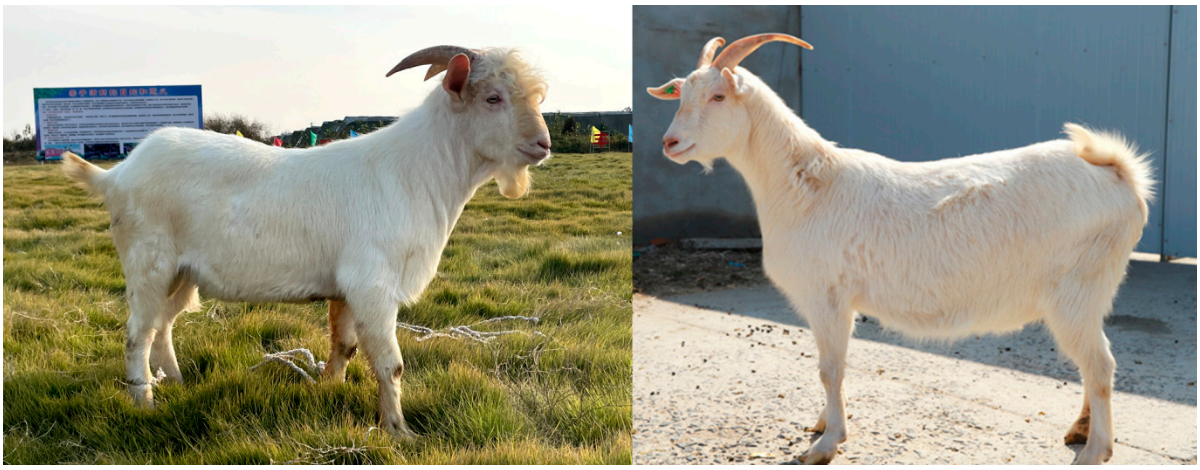

Figure. S1. Qianqiu Goat photo (left for male; right for female)

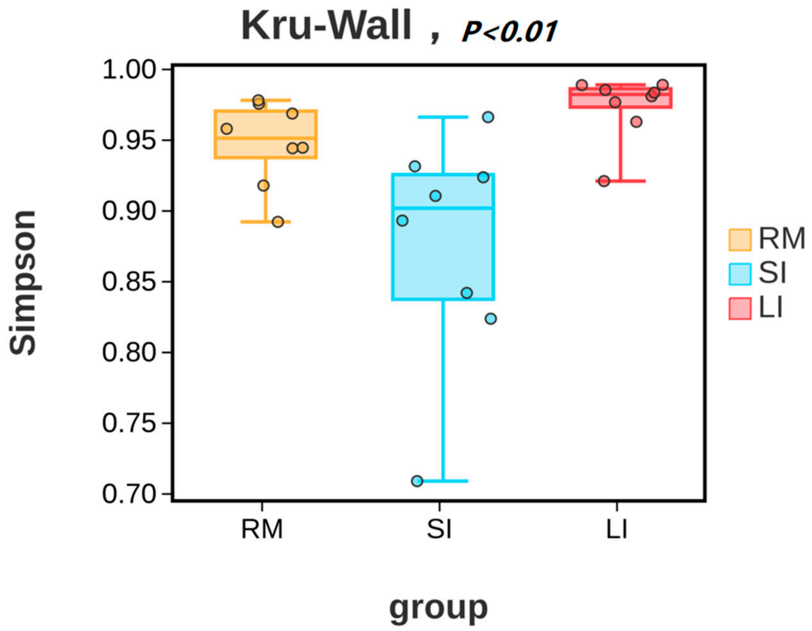

Figure. S2. Simpson diversity index of bacterial communities in chyme samples from the three GI regions

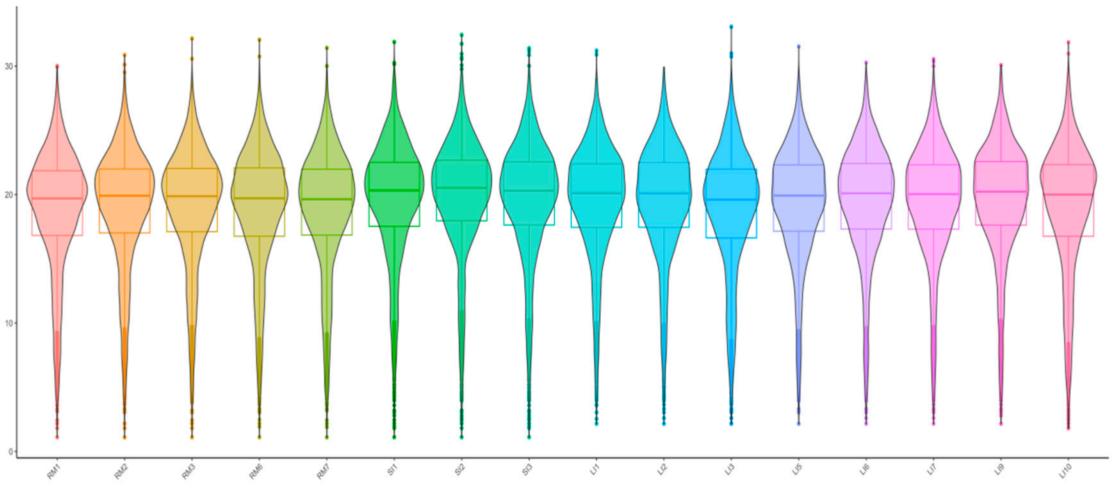

Figure. S3. Sample violin plot

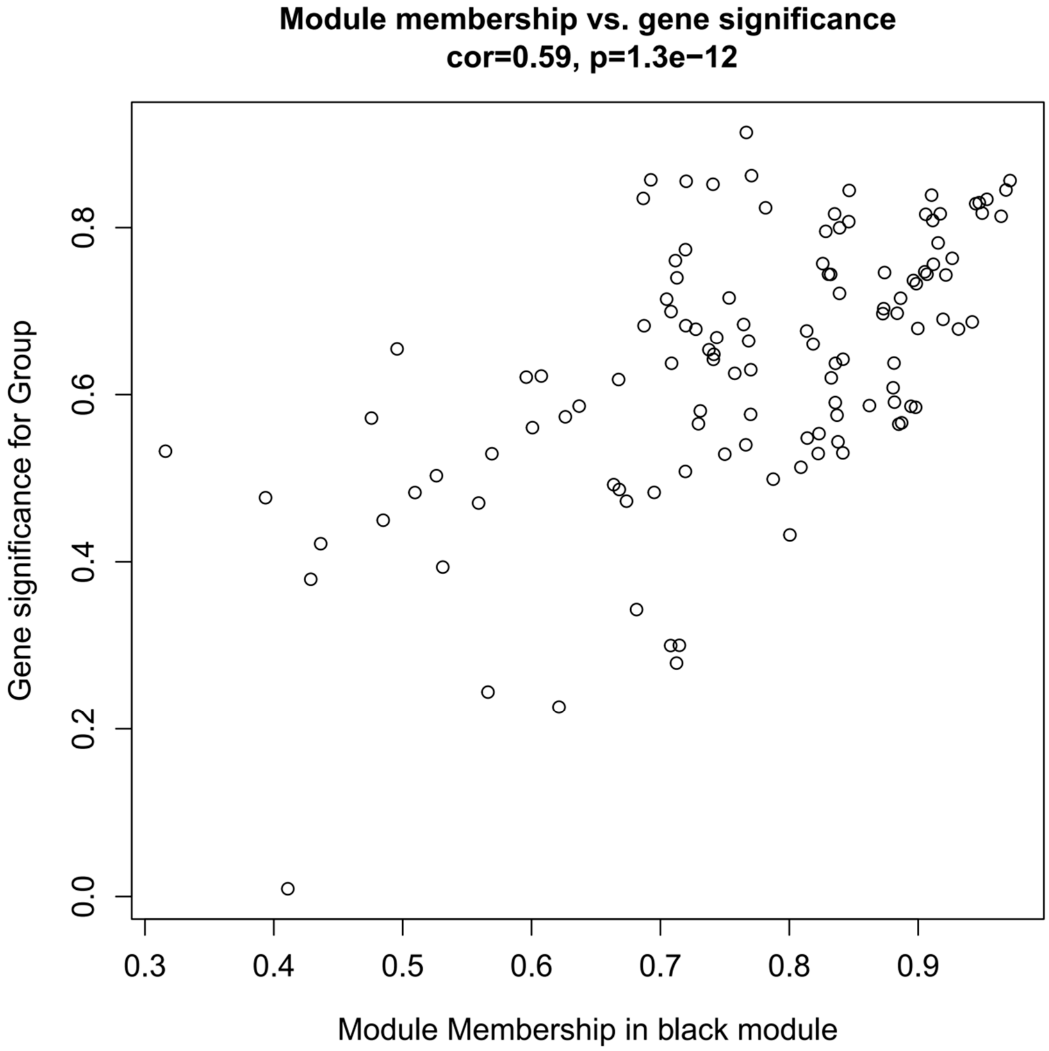

Figure. S4. Correlation scatter plot between Black module membership (MM) and tissue metabolite significance (GS).

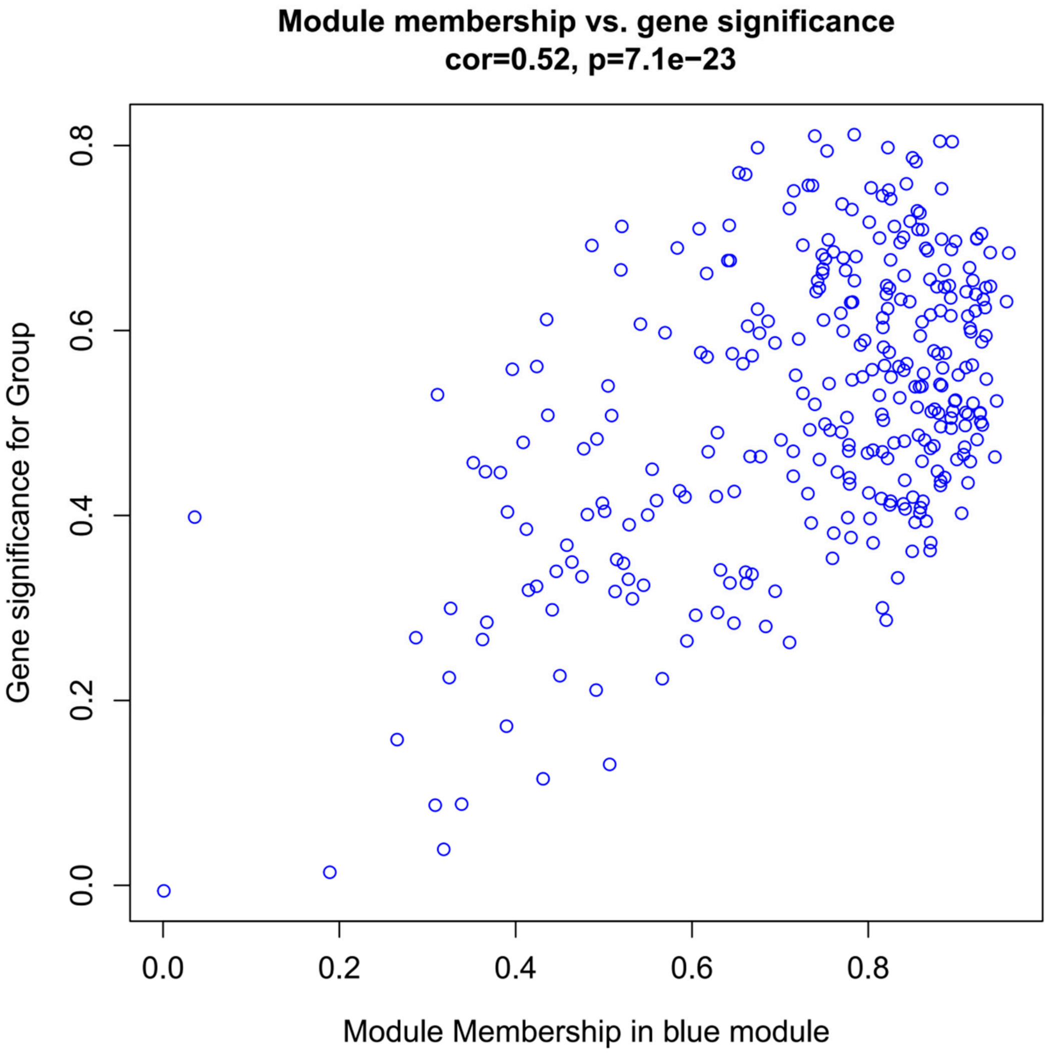

Figure. S5. Correlation scatter plot between Blue module membership (MM) and tissue metabolite significance (GS).

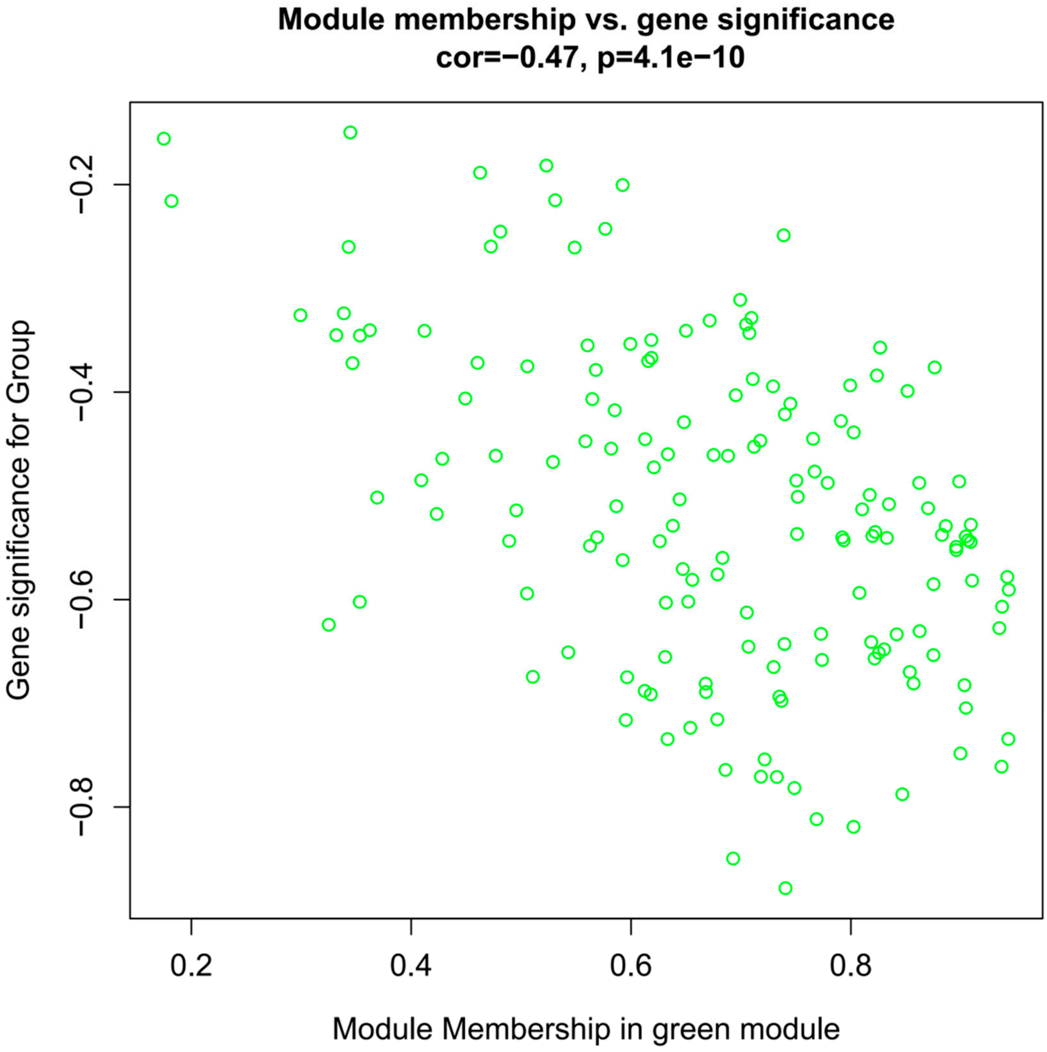

Figure. S6. Correlation scatter plot between Green module membership (MM) and tissue metabolite significance (GS).

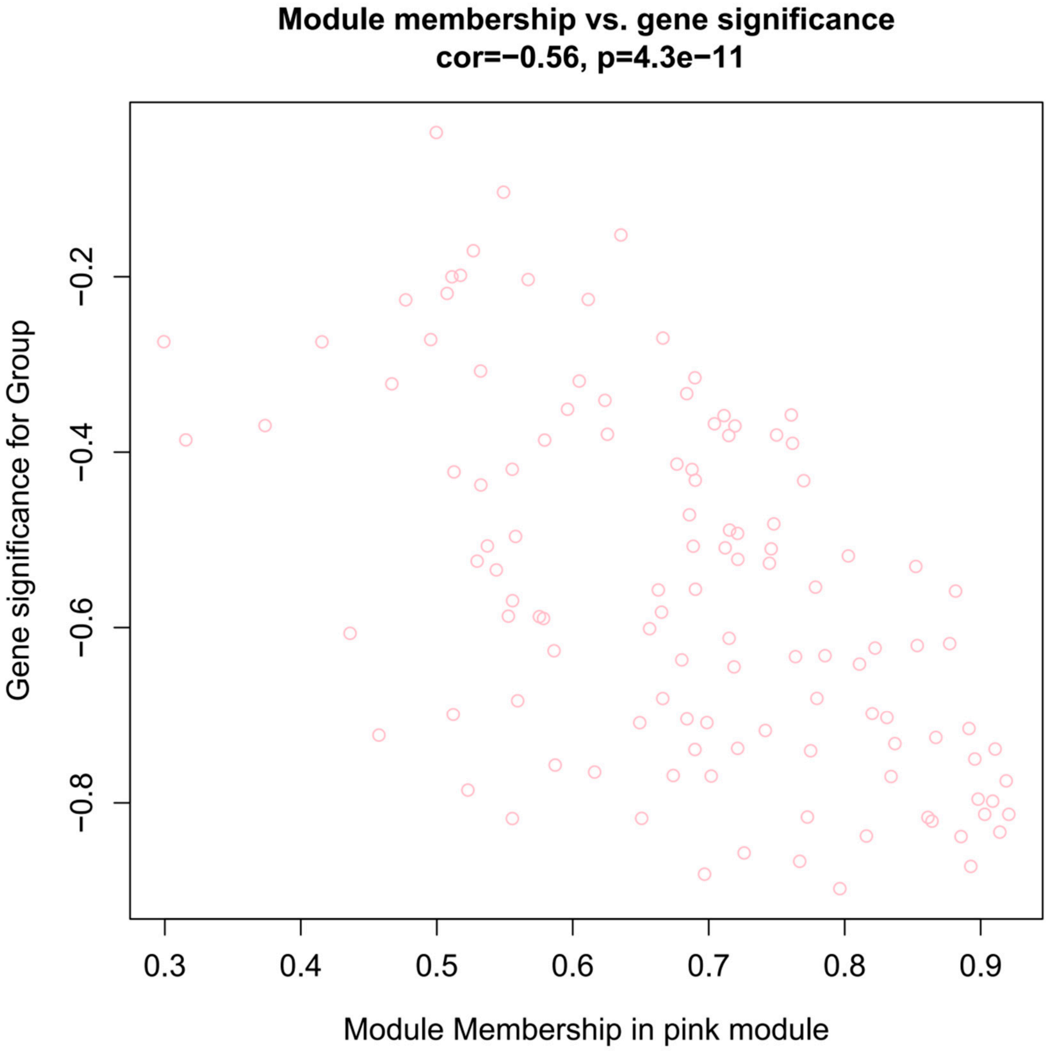

Figure. S7. Correlation scatter plot between Pink module membership (MM) and tissue metabolite significance (GS).

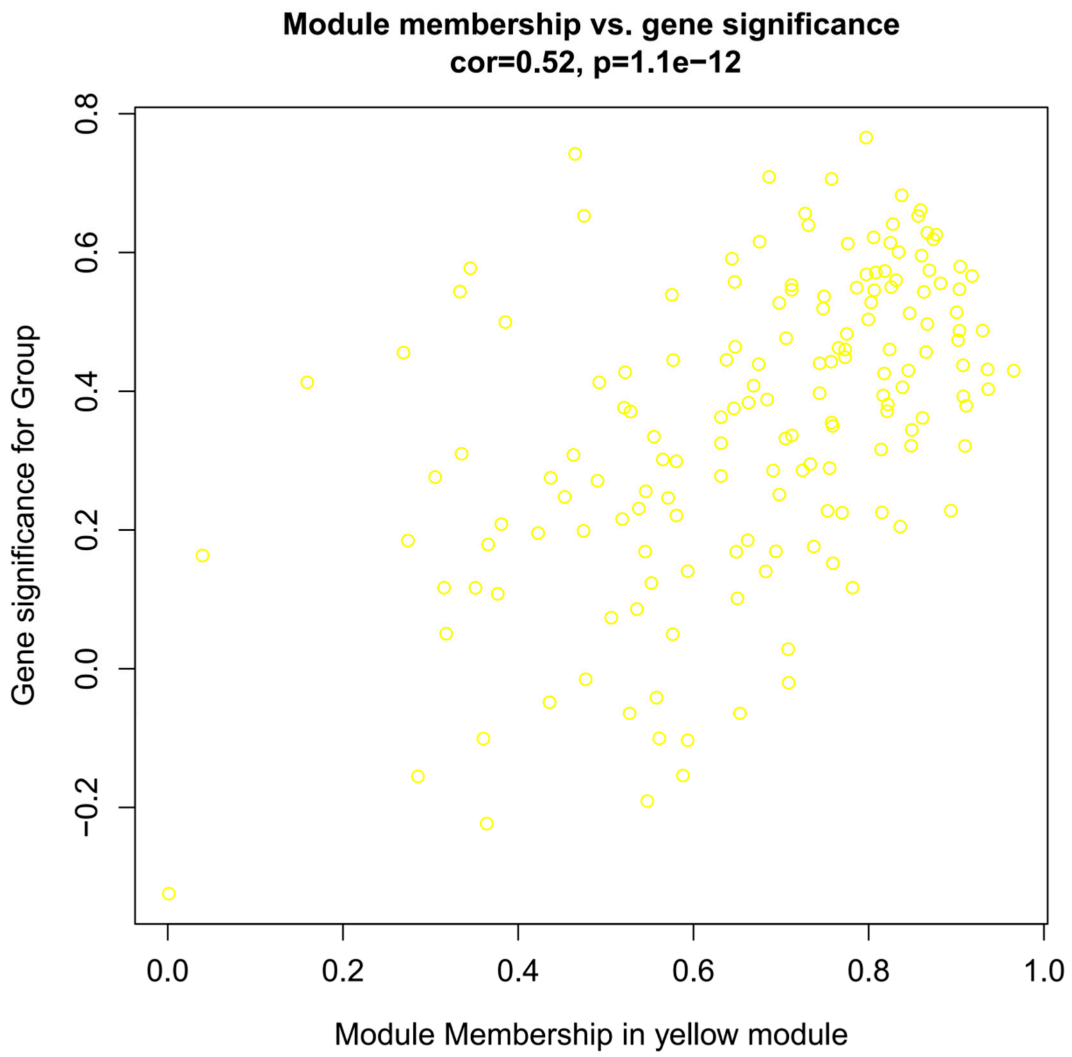

Figure. S8. Correlation scatter plot between Yellow module membership (MM) and tissue metabolite significance (GS).

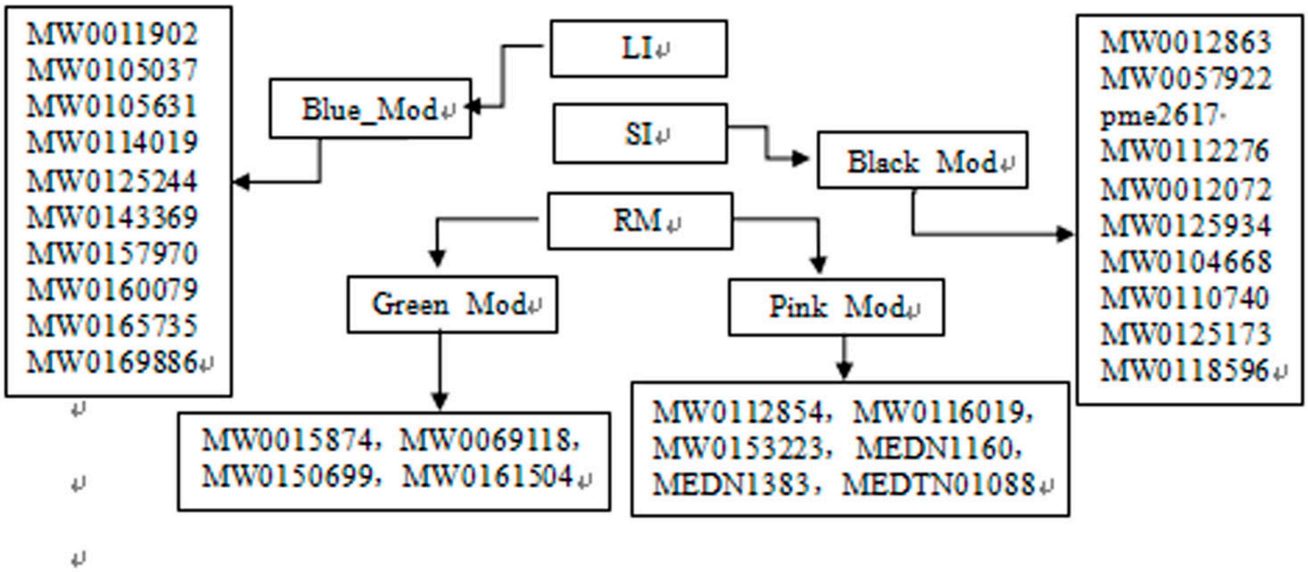

Figure. S9. Tissue-specific metabolic features (For each tissue, the ten metabolites with the highest Gene Significance (GS) scores were identified as hub metabolites indicative of tissue-specific metabolic signatures
